# Supplementary figures and images for: Defective nucleotide-dependent assembly and membrane fusion in Mfn2 CMT2A variants improved by Bax
Source: Life Sci Alliance. 2020 Apr 3;3(5):e201900527. doi: 10.26508/lsa.201900527 (PMC7136618; doi:10.26508/lsa.201900527)

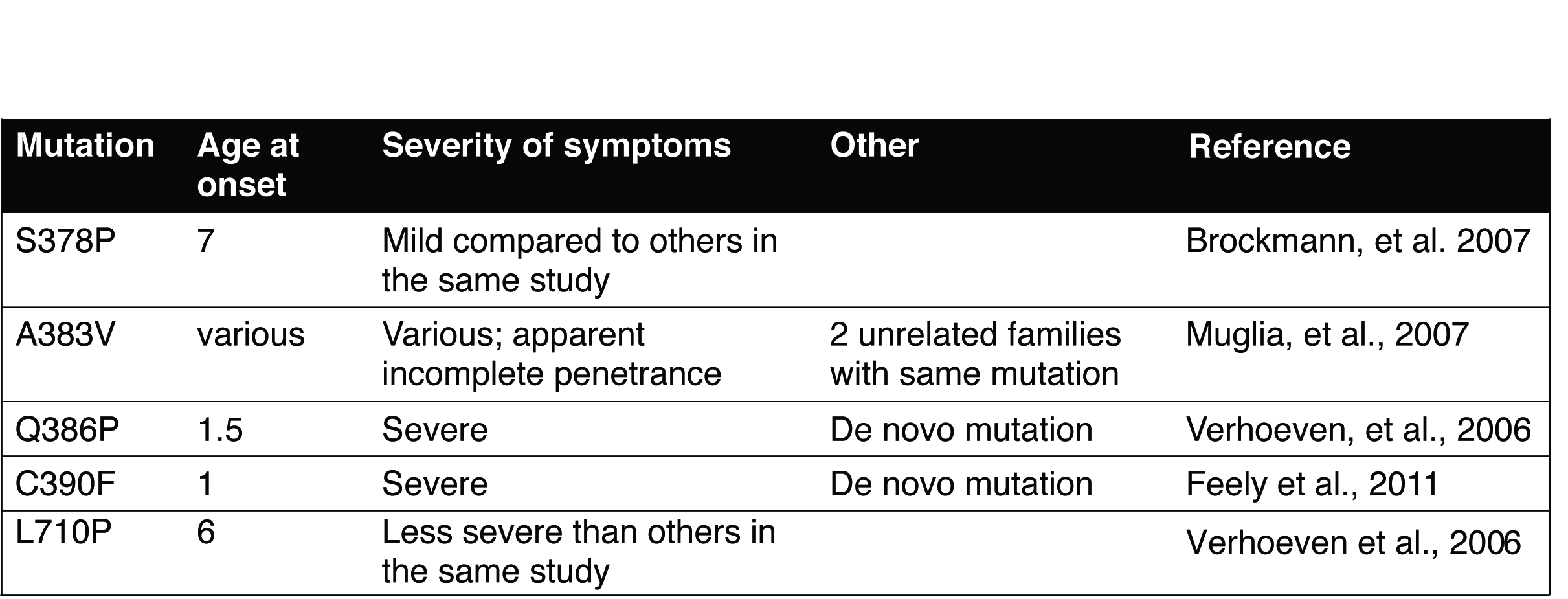

Supplement: Supplementary file 1 [file LSA-2019-00527_TableS1.tif]
